# Zoonotic transmission of the *Mycobacterium tuberculosis* complex between cattle and humans in Central Ethiopia

**DOI:** 10.3389/fvets.2025.1527279

**Published:** 2025-03-10

**Authors:** Tefera Woldemariam, Temesgen Mohammed, Aboma Zewude, Mahlet Chanyalew, Hazim O. Khalifa, Gezahegne Mamo, Gobena Ameni

**Affiliations:** ^1^School of Veterinary Medicine, Hawassa University, Hawassa, Ethiopia; ^2^College of Agriculture and Veterinary Medicine, United Arab Emirates University, Al Ain, United Arab Emirates; ^3^Aklilu Lemma Institute of Pathobiology, Addis Ababa University, Addis Ababa, Ethiopia; ^4^Department of Pharmacology, Faculty of Veterinary Medicine, Kafrelsheikh University, Kafr El-Sheikh, Egypt; ^5^College of Veterinary Medicine and Agriculture, Addis Ababa University, Debre Zeit, Ethiopia

**Keywords:** Ethiopia, reverse zoonosis, tuberculosis, zoonosis, Central Ethiopia

## Abstract

**Introduction:**

The zoonotic transmission of tuberculosis (TB) from cattle to humans has long been recognized, while the reverse transmission from humans to animals has only recently been reported. The socioeconomic conditions in rural Ethiopia are conducive to the zoonotic and reverse zoonotic transmission of TB between cattle and humans. This study aimed to explore the transmission of the *Mycobacterium tuberculosis* complex between cattle and humans in Central Ethiopia.

**Methods:**

To achieve this objective, a cross-sectional study was conducted involving 1,896 cattle slaughtered at two abattoirs and 392 human subjects suspected of tuberculosis who visited health institutions for treatment. Mycobacteriological culture and spoligotyping were used for the study. Additionally, the Spoligotype International Types and VNTR (variable number of DNA tandem repeats) International Types (SITVIT2) database and the online tool “Run TB-Lineage” were used to identify SIT and lineages of the isolates from both humans and cattle.

**Results:**

Culture positivity was found in 26.3% (21/80) of the gross TB-suspicious tissue lesions in the lungs and lymph nodes (mandibular, retropharyngeal, cranial, and caudal mediastinal, as well as left and right bronchial, hepatic, and mesenteric lymph nodes) of cattle. Of the 21 cattle isolates, 12 (57.2%) were identified as *M. bovis,* while the remaining 9 (42.8%) were classified as *M. tuberculosis*. Similarly, only 22% (86/392) of the sputum samples from TB-suspicious humans were culture positive. These 86 human isolates included 81 *M. tuberculosis*, three *M. africanum,* and two *M. bovis, as determined* by spoligotyping. SIT50, SIT118, and SIT1318, which belong to the human species, were isolated from both humans and cattle. The two human *M. bovis* isolates exhibited the pattern of SB1443, which was not identified in cattle within this study area.

**Conclusion:**

The zoonotic and reverse zoonotic transmissions of TB were confirmed in Ethiopia by isolating two *M. bovis* from humans and nine *M. tuberculosis* from cattle, which suggested a greater role for *M. tuberculosis* in cattle compared to *M. bovis* in humans.

## 1 Introduction

Bovine tuberculosis (bTB) is a chronic disease affecting animals, characterized by the progressive development of granulomatous lesions or tubercles in the lymph nodes, lung tissue, and other organs. This disease is primarily caused by *Mycobacterium bovis*, a member of the *Mycobacterium tuberculosis* (*M. tuberculosis*) complex, and to a lesser extent by *M. tuberculosis*. The disease leads to significant economic losses for the agriculture sector and poses public health challenges by causing zoonotic TB in humans. According to the World Organization for Animal Health, 82 (44%) of the 188 countries and territories reported the presence of bTB between January 2017 and June 2018 (1). Thus, this disease occurs worldwide, although most developed countries have reduced or eliminated it from their cattle populations, except in limited areas where wild animals, as reservoirs of the infection, interfere with control programs. Conversely, the disease remains prevalent in many African countries and parts of Asian countries (1–4). The prevalence of bTB in animal populations in developing countries can be attributed to insufficient or ineffective control measures that address the socioeconomic issues related to the current test-and-slaughter approach (3).

The direct correlation between *M. bovis* infection in cattle and zoonotic TB in the human population has been well documented in many industrialized countries (3). Zoonotic TB, caused by *M. bovis,* is transmitted from animals to humans through the consumption of contaminated foods and the inhalation of cough droplets from infected animals during close physical contact. It also poses an occupational hazard for farmers, slaughterhouse workers, butchers, and veterinary professionals.

Epidemiological studies of TB in cattle and humans in Zambia (5) and Ethiopia (6) indicated associations between tuberculin-positive cattle and human TB. In Zambia, it was observed that households reporting a TB case within the last year were seven times more likely to own herds containing tuberculin-positive cattle. Similarly, tuberculin positivity was three times higher in cattle owned by farmers with active TB than in those owned by farmers without active TB in Ethiopia. These observations suggest potential transmission of mycobacteria between humans and cattle, which could involve the transmission of *M. bovis* from cattle to humans or the transmission of *M. tuberculosis* from humans to cattle. A few studies in Ethiopia reported the isolation of *M. bovis* from sputum and fine needle aspirates of human TB cases (7, 8). According to a previously published study (9), in countries where bTB is still common and pasteurization of milk is not practiced, an estimated 10–15% of human TB is caused by *M. bovis,* with infections believed to occur through drinking raw milk (10) and through inhalation in rural areas among people living in close contact with cattle (11).

The limited number of studies conducted in Ethiopia lacks extensive geographic coverage and representativeness. Geographic regions such as the East Shewa Zone of central Ethiopia, where dairy cattle and beef farming are commonly practiced, have not been considered. The East Shewa Zone is at risk for mycobacteria transmission between cattle and humans. Numerous butchers in the area slaughter cattle from beef farms and nearby rural areas to sell raw meat, making it well-known for this business. In addition to beef cattle, farmers in the surrounding towns and rural districts also keep dairy cows. A significant percentage of raw milk is supplied to consumers in the area. These common practices of consuming raw milk and raw meat could facilitate the transmission of *M. bovis* from cattle to humans. Furthermore, Ethiopia ranks seventh among the 20 countries with the highest burden of human TB, with an incidence rate of 165 cases per 100,000 population (12).

East Shewa Zone is one of the most densely populated areas of Ethiopia, characterized by a high rate of human mobility, as the main road to the Port of Djibouti passes through the Zone. Therefore, there are favorable conditions for the transmission of human TB, and there is a significant risk of *M. tuberculosis transmission* from humans to cattle due to close physical contact between them and their cattle. It is essential to investigate whether transmission of TB occurs between cattle and humans, given these conducive epidemiological conditions. The present study was conducted to examine the zoonotic transmission of the *Mycobacterium tuberculosis* complex between cattle and humans in central Ethiopia.

## 2 Materials and methods

### 2.1 Study setting

The study was conducted from 2018 to August 2020 in the towns of Adama and Bishoftu, located within the East Shewa Zone of Oromia Regional State in Central Ethiopia. Bishoftu and Adama are situated 47 km and 95 km southeast of Addis Ababa, respectively. These two towns are known for their beef production and the sale of beef meat. Cattle from the beef farms and rural areas surrounding these towns are slaughtered in local abattoirs, and raw meat consumption is quite common in the region. In addition to beef cattle, dairy cows are raised by farmers living in these towns and the surrounding rural districts. Consequently, a significant percentage of raw milk is supplied to consumers in these towns and nearby districts. Unfortunately, BTB is prevalent in the country’s dairy farms, which are not expected to be free from the disease.

### 2.2 Study design

A cross-sectional study design was used to evaluate the transmission of *M. tuberculosis* complex species between cattle and humans in Central Ethiopia.

### 2.3 Sample size

Sample size determination was based on the availability of funds and logistical support for the project’s research activities. Since the main objective of this research was to evaluate the transmission of *M. tuberculosis* between cattle and humans, efforts were made to collect the maximum number of samples by examining a large number of cattle and gathering a significant number of sputum samples. No statistical calculations were used to estimate the sample size in either the human or cattle studies. Thus, the available funds and logistics facilitated the examination of 1,896 cattle at two abattoirs (1,266 at Adama and 630 at Bishoftu). Additionally, 392 sputum samples (274 from Adama and 118 from Bishoftu) were collected from the two towns.

### 2.4 Participants of the study

The study subjects comprised cattle slaughtered at these two abattoirs and human TB patients who visited healthcare centers in the two towns during the study period. A postmortem examination for BTB was conducted on cattle from beef farms located in either of the two towns or the surrounding districts. Similarly, sputum samples from individuals living in these towns or the surrounding districts with connections to either dairy or beef cattle were used for bacteriological and molecular analysis.

### 2.5 Variables

The primary outcome was the isolation of the same species of *M. tuberculosis* complex from both cattle and humans, suggesting possible TB transmission between the two. Other secondary outcomes included the percentage of cattle with TB lesions in their tissues and the percentage of suspicious TB lesions that tested positive via culture and spoligotyping. In the human study, the secondary outcome focused on the percentage of presumptive patients who tested positive through culture and spoligotyping. Diagnostic criteria for the cattle study included gross BTB lesions confirmed by culture and spoligotyping. For the human study, the diagnostic criteria included clinical examinations such as persistent coughing for more than 2 weeks, chest pain, hemoptysis, night sweats, and weight loss, followed by confirmation through sputum culturing and spoligotyping of the cultured isolates. Individuals showing signs and symptoms of TB, confirmed by sputum culturing, were treated with anti-TB drugs. The final confirmation of probable transmission of *M. tuberculosis* complex species between cattle and humans was achieved through spoligotyping.

### 2.6 Sample collection

The primary objective of the study was to confirm the transmission of the *Mycobacterium tuberculosis* complex between cattle and humans. Focus was placed on searching for BTB lesions in the tissues of cattle, followed by the collection of suspicious tissue lesions. The tissues with detected suspicious lesions were recorded. BTB suspicious tissue samples were collected in universal bottles containing normal saline and transported under cold chain conditions to the TB laboratory at the Aklilu Lemma Institute of Pathobiology, Addis Ababa University.

A total of 1,896 cattle were examined at two abattoirs, from which 80 BTB suspicious tissue lesions were collected from the lungs and lymph nodes (mandibular, retropharyngeal, cranial, and caudal mediastinal, left and right bronchial, hepatic, and mesenteric lymph nodes) for culturing. The number of animals recruited was determined by logistical availability and the convenience of the slaughterhouses. Human sample collection was conducted as part of routine diagnostic procedures for TB. Sputum samples were submitted to hospitals and health centers by 392 TB-suspicious cases. Consequently, 274 sputum samples were collected from Adama Town, while 118 sputum samples were collected from Bishoftu Town. The sputum samples were transported under cold chain conditions to the TB laboratory at the Aklilu Lemma Institute of Pathobiology, Addis Ababa University, for culturing.

### 2.7 Post-mortem examination and tissue sampling

A postmortem examination was conducted following the procedure described by (13). Each of the seven lobes of the lungs was thoroughly inspected and palpated for any suspicious gross TB lesions. Similarly, the mandibular, retropharyngeal, cranial and caudal mediastinal, left and right bronchial, hepatic, and mesenteric lymph nodes were sliced into 2 mm sections and subsequently examined for the presence of visible lesions, as per the protocol described earlier (14, 15). Tissues showing macroscopic lesions compatible with bTB were collected in a universal bottle with a 0.9% saline solution and transported to the laboratory under cold chain conditions. A total of 80 suspicious lesions were collected from the 1,896 cattle investigated and processed for culturing, as detailed below.

### 2.8 Culturing of suspicious tissue lesions

Samples from suspected TB lesions were further processed to isolate mycobacteria according to the protocols of the World Organization for Animal Health (16). Tissue specimens for culture were collected in sterile universal bottles containing 5 mL of a 0.9% saline solution. They were then transported in an icebox at a stable temperature of 4°C to the Aklilu Lemma Institute of Pathobiology. In the laboratory, the specimens were sectioned with sterile blades and homogenized using a mortar and pestle. The homogenate was decontaminated by adding an equal volume of 4% NaOH and centrifuging at 1,865 g for 15 min. The supernatant was discarded, and the sediment was neutralized with 1% (0.1 N) HCl, using phenol red as an indicator. Neutralization was considered to have been achieved when the solution’s color changed from purple to yellow. Subsequently, 0.1 mL of suspension from each sample was spread over a slant of Lowenstein-Jensen medium. Duplicate slants were used, one enriched with sodium pyruvate and the other with glycerol. Cultures were incubated aerobically at 37°C for at least eight weeks, with weekly observations of colony growth.

### 2.9 Culturing of sputum samples

Sputum samples were collected and processed for culture following the WHO guidelines (17). Briefly, an equal volume of 4% NaOH was mixed with the sputum sample, and the mixture was centrifuged at 3,000 rpm for 15 min at room temperature. After decanting the supernatant, the sediment was neutralized with 2 N HCl using phenol red as an indicator. Neutralization was achieved when the solution changed color from purple to yellow. Thereafter, 100 μL of the suspension was inoculated onto two sterile LJ medium slopes, which were enriched with either pyruvate or glycerol. The inoculated media were then incubated at 37°C in a slanted position for one week and in an upright position for four to five weeks. Bacterial growth was monitored weekly until the 8th week of culture.

### 2.10 Preparation of DNA for molecular typing

The colonies were harvested from the surface of the LJ medium and re-suspended in 200 μL of sterile double-distilled water. Subsequently, the colonies and water were mixed thoroughly. The mixture was then heated to 80°C for 50 min in a water bath to release mycobacterial DNA, which was used for subsequent spoligotype analysis.

### 2.11 Spoligotyping of mycobacterial isolates

Spoligotyping was performed on 107 *M. tuberculosis* complex isolates at the Aklilu Lemma Institute of Pathobiology, Addis Ababa University, following the standard operating procedure that was used by (18) and primarily developed by (19). The DNA released by heat-killing of the colonies was used as a template to amplify the direct repeat (DR) region of *M. tuberculosis* complex by polymerase chain reaction (PCR) using oligonucleotide primers derived from the DR sequence, RDa (5’GGTTTTGGGTTTGAACGAC3’) and RDb (5’CCGAGAGGGGACG GAAAC3’) primers (19). The total volume of the PCR reaction mixture was 25 μL, consisting of 12.5 μL of HotStarTaq Master Mix (Qiagen; providing a final concentration of 1.5 mM MgCl2 and 200 mM of each deoxyribonucleotide triphosphate), 2 μL of each primer (20 pmol each), 5 μL of a suspension of DNA template (approximately 10–50 ng), and 3.5 μL of Qiagen water. The mixture was heated for 15 min at 96°C and then subjected to 30 cycles of 1 min at 96°C, 1 min at 55°C, and 30 s at 72°C, followed by a final extension at 72°C for 10 min.

Immediately before running the spoligotyping, the PCR product was denatured in a thermocycler at 96°C for 10 min and then removed from the thermocycler and placed on ice to prevent further denaturation of the PCR products. The denatured PCR product was subsequently loaded onto a membrane covalently bonded with a set of 43 oligonucleotides, each corresponding to a unique spacer DNA sequence within the DR locus of the *M. tuberculosis* complex, and hybridized at 60^o^c for 1 h. After hybridization, the membrane was washed twice for 10 min in 2x SSPE (1x SSPE consists of 0. 18 M NaCl, 10 mM NaH_2_ PA_4_, and 1 mM EDTA [pH 7. 7]) with 0.5% sodium dodecyl sulfate at 60^o^C and then incubated in a 1: 4000 dilution of streptavidin peroxidase (Boehringer) for 1 h at 42°C. The membrane underwent two additional washes for 10 min in 2 x SSPE with 0.5% sodium dodecyl sulfate at 42°C and was rinsed with 2 x SSPE for 5 min at room temperature. Hybridized DNA was detected using the enhanced chemiluminescence method (Amersham, Biosciences, Amersham, UK) and exposed to X-ray film (Hyperfilm ECL, Amersham). A mixture of 10 mL of ECL reagent 1 and 10 mL of ECL reagent 2 was prepared and added to the membrane, which was rinsed in the solution for 5 min at room temperature. The membrane was then attached to a film in the darkroom, placed in a cassette, and incubated for 15 min at room temperature. After incubation, the film was removed and placed in a developer solution for 2 min. The film was subsequently rinsed with tap water for 15 s and placed in a fixer solution for 1 min. Finally, the film was dried and used for result interpretation. The presence of a spacer was indicated as a black square, while its absence was indicated as a white square on the film. Subsequently, the black squares were converted to 1, while the white squares were converted to 0 and transferred to the Spoligotype International Types-VNTR International Types (SITVIT) database for identifying the Spoligotype International Types (SIT) and the lineages of the isolates.

### 2.12 Data analysis

Proportions were used to indicate the number of cattle with TB lesions. Tissue lesions and sputum culture positivity were presented as proportions. In the molecular aspect of the study, SIT numbers and lineages of isolates were identified using the SITVIT1 Database and Run TB-Lineage. The results of spoligotyping were converted into octal and binary formats and then submitted into the query box, allowing the retrieval of the strain names from the database if the spoligotype pattern of the strain matched one already registered in the SPolDB4 database, accessible at http://www.pasteur-guadeloupe.fr:8081/SITVITDemo/ (SITVIT1 Database) (20). If the strain’s pattern had not been previously registered in the database, it was classified as an orphan. The lineages were also generated by entering binary and octal formats into the query box of the SITVIT1 Database and Run TB-Lineage.

## 3 Results

### 3.1 Tissue culture positivity

Gross TB lesions were detected in 80 out of 1,896 (4.22%) of the cattle slaughtered at the Bishoftu and Adama abattoirs. These lesions were observed in various tissues of the lungs and lymph nodes, including the mandibular, retropharyngeal, cranial, and caudal mediastinal, left and right bronchial, hepatic, and mesenteric lymph nodes. They were more frequent and severe in the lungs and thoracic lymph nodes, followed by those in the head and neck. Accordingly, about 21 out of 80 (26.3%) of the suspected tissue lesions tested positive for mycobacterial growth.

### 3.2 Sputum culture positivity and demographic characteristics of the study subjects

Table 1 shows selected demographic characteristics of human TB-suspicious cases, which were used as a source of sputum from the two towns. The sputum culture positivity rate was 21.9% (86/392). Fifty culture-positive samples originated from Adama, while 36 were from Bishoftu. The culture positivity rate for samples collected in Adama Town was 18.2% (50/274), whereas it was 30.5% (36/118) for those collected in Bishoftu Town.

**Table 1:** Selected demographic characteristics of the study participant’s and their association with culture positivity.

Category	Number (%) positive	Total	*χ*^2^ (95% CI)	*p*-value
Adama Town				
Age				
Group1:10–29	12 (10.3%)	116	6.48 (2.8,20.8)	*p* < 0.05
Group2:30–59	31 (22.3%)	139	9.40 (7.5,49.00)	*p* < 0.01
Group3: ≥60	7 (36.8%)	19	1.9 (−4.8,37.5)	*p* > 0.05
Total	50	274		
Sex				
Female	25 (18.4%)	136	0.0 (−8.9,9.5)	*p* > 0.05
Male	25 (18.1%)	138		
Total	50	274		
Residence				
Rural	34 (18.5%)	184	0.32 (−6.9,14.8)	*P* > 0.05
Urban	16 (21.6%)	90		
Total	50	274		
Bishoftu Town				
Age				
Group1:10–29	15 (31.9%)	48	0.68 (−9.7,24.3)	*p* > 0.05
Group2:30–59	14 (24.6%)	58	1.5 (−9.1,44.0)	*p* > 0.05
Group3: ≥60	7 (50%)	14	3.38 (−1.2,50.5)	*p* > 0.05
Total	36	120		
Sex				
Female	20	63	0.17 (−13.0,19.5)	*p* > 0.05
Male	16	57		
Total	36	120		
Residence				
Rural	10	46	2.34 (−3.8,28.4)	*p* > 0.05
Urban	26	74		
**Total**	**36**	**120**		

### 3.3 Molecular identification of species and strains of mycobacteria

#### 3.3.1 Animal isolates

Spoligotyping of 21 isolates from cattle yielded 18 different spoligotype patterns. Of the 21 isolates, 12 (57.2%) were confirmed to be *M. bovis,* while the remaining nine (42.8%) were *M. tuberculosis.* The isolates were grouped into 3 clustered strains, each containing 2 isolates (28.63%), and 15 (71.4%) single isolates (Table 2). Out of the 18 spoligotype patterns, 6 were registered in the international database, while the remaining 12 were not found in it. The 12 strains of *M. bovis* isolated from cattle were represented by 9 different spoligotype patterns. Of these 9 clusters of *M. bovis*, 7 were new and had not been previously reported in the *M. bovis* spoligotype database, while the remaining two belonged to spoligotypes SB1265 (two isolates) and SB1176 in the database *M.bovis*.org. The nine strains of *M. tuberculosis* isolated from cattle represented 9 different spoligotype patterns. Of these nine clusters, five clusters were not registered in the international spoligotype SITVIT2 database and were thus designated as orphan strains, while the remaining four clusters corresponded to spoligotypes SIT 50, 118, 117, and 1,318 in the SITVIT2 database, with the majority belonging to the Euro-American lineage.

**Table 2:** Spoligotype patterns of *M. bacterium* tuberculosis complex species isolated from cattle in Central Ethiopia.

SIT/SB	Isolates with similar pattern	CBN* lineage	SITVIT2 lineage	Octal number	Binary
SB1176	1	*Mycobacterium bovis*	BOV_1	602,773,761,000,200	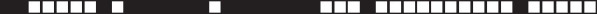
SB1256	2	*Mycobacterium bovis*	BOV_1	616,763,777,777,600	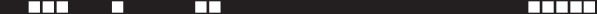
SIT50	1	Euro-American	H3	777,777,777,720,771	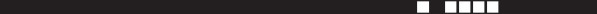
SIT118	1	Euro-American	T2	777,767,777,760,771	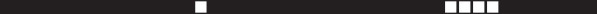
SIT117	1	Euro-American	T1	777,767,777,760,731	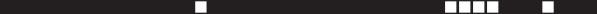
SIT1318	1	Euro-American	T1	577,767,777,760,771	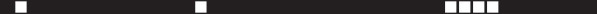
Orphan	1	*Mycobacterium bovis*	BOV	416,743,777,756,600	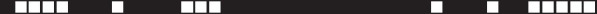
Orphan	1	*Mycobacterium bovis*	BOV_1	612,763,761,000,200	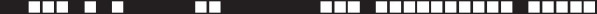
Orphan	1	*Mycobacterium bovis*	BOV_1	612,773,761,000,200	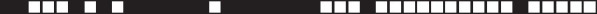
Orphan	1	*Mycobacterium bovis*	BOV_1	400,201,434,200,000	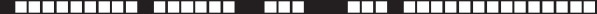
Orphan	1	*Mycobacterium bovis*	BOV_1	402,773,761,000,200	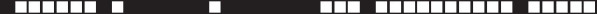
Orphan	2	*Mycobacterium bovis*	BOV_1	416,763,577,756,600	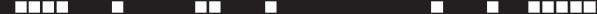
Orphan	2	*Mycobacterium bovis*	BOV_1	612,773,761,000,200	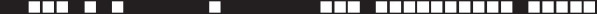
Orphan	1	Euro-American	H3	577,767,777,700,771	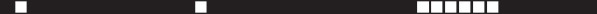
Orphan	1	Indo-Oceanic	MANU1	777,767,777,777,771	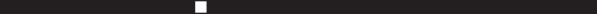
Orphan	1	Euro-American	T	775,767,577,760,771	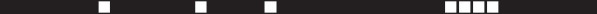
Orphan	1	Euro-American	T	777,767,777,760,661	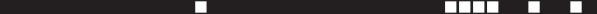
Orphan	1	Euro-American	X2	417,763,575,600,200	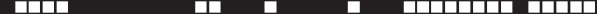

#### 3.3.2 Human isolates

Spoligotyping of 86 isolates from humans yielded 35 different spoligotype patterns. The isolates were grouped into 14 clustered strains, comprising 65 (75.6%) isolates and 21 (24.4%) single strains (Table 3). The overall diversity among the isolates was 40.7%. Out of the 35 spoligotype patterns, 27 were recorded in the international database, while the remaining eight were not found in the database. The most frequently identified strains were SIT 149, SIT 53, and SIT 118, representing 18, 11, *and 6* isolates, respectively, and belonging to the *M. tuberculosis* species. Classification of the spoligotype patterns using TB-insight RUN TB-Lineage showed that the Euro-American (E-A) lineages dominated, comprising 87.2%, while 3.5% of the isolates were classified as Central-Asian (CAS) lineages, another 3.5% as *M. africanum* lineages, and 1.2% as Indo-oceanic lineages. Two *M. bovis* isolates (2.3%) were identified by their characteristic spoligotype features, which include missing spacers 3, 9, 16, and 39–43. Among the unregistered patterns, four strains were attributed to the E-A lineage, three to *M. africanum*, one to CAS, and one to the Indo-oceanic lineage. The two *M. bovis* strains isolated from sputum had spoligotypes SB01443 in the *M.bovis*.org database.

**Table 3:** Spoligotype patterns of *Mycobacterium tuberculosis* complex species isolated from humans in Central Ethiopia.

SIT	Isolates with similar pattern	CBN* lineage	SITVIT2 sublineage	Octal number	Binary
149	18	EA	T3 ETH	777,000,377,760,771	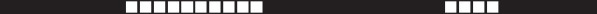
777	3	EA	H4	777,777,777,420,771	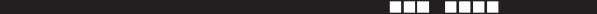
336	2	EA	X1	777,776,777,760,731	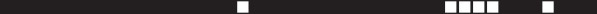
53	11	EA	T1	777,777,777,760,771	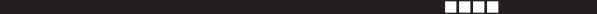
50	2	EA	H3	777,777,777,720,771	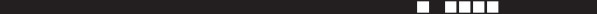
134	3	EA	H3	777,777,777,720,631	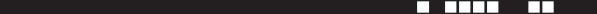
1745	1	EA	T3	773,737,777,760,771	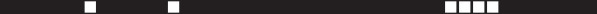
245	1	EA	T1	777,777,777,760,671	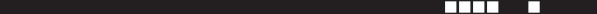
37	4	EA	T3	777,737,777,760,771	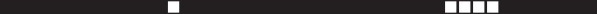
221	1	EA	X1	777,766,777,760,771	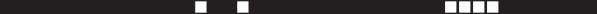
1,221	1	EA	T1	757,767,777,760,771	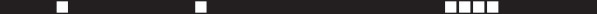
1,547	4	EA	T3	777,727,777,760,771	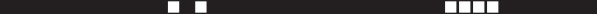
51	1	EA	T	777,777,777,760,700	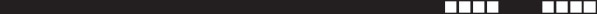
75	1	EA	H3	777,767,777,720,771	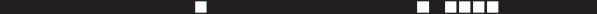
7	1	EA	T1	377,777,777,760,771	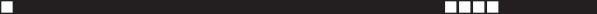
118	6	EA	T2	777,767,777,760,771	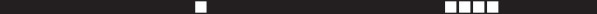
1,318	3	EA	T1	577,767,777,760,771	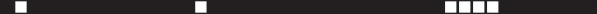
117	3	EA	T1	777,767,777,760,731	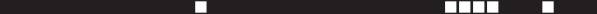
1,687	1	EA	T1	777,767,775,760,771	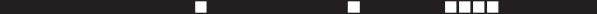
1802	1	EA	H3	777,767,777,720,631	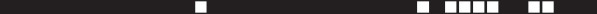
611	1	EA	T1	777,767,777,560,771	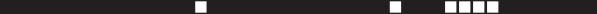
834	1	EA	U	777,767,777,740,771	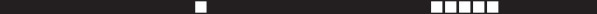
116	1	EA	H3	777,767,775,720,771	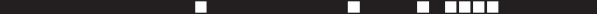
Orphan	1	EA	H3	477,767,777,720,571	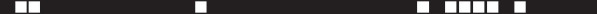
Orphan	1	EA	X2	777,737,777,760,000	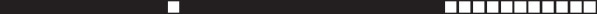
Orphan	1	EA	T	755,777,777,760,671	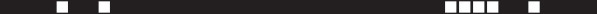
Orphan	1	EA	X2	777,727,777,760,000	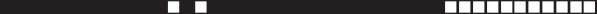
309	1	EAI	CAS1	703,767,740,003,171	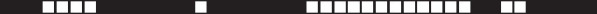
25	1	EAI	CAS1	703,777,740,003,171	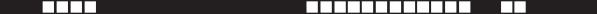
141	2	CAS	MTB CAS	703,767,740,003,771	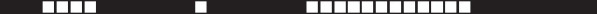
Orphan	1	CAS	CAS	703,777,400,002,061	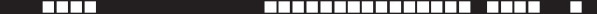
Orphan	2	*Mycobacterium africanum*	Manu2	575,347,777,763,661	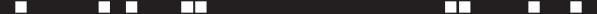
Orphan	1	*Mycobacterium africanum*	X2	554,377,777,760,660	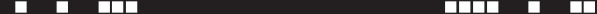
Orphan	1	IO	CAS	713,600,000,001,171	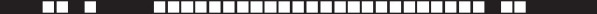
1,443	2	*Mycobacterium bovis*	BOV	616,777,777,777,600	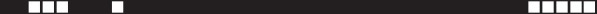

## 4 Discussion

In the present study, *M. tuberculosis* complex species were isolated from 86 human TB patients at Bishoftu and Adama Referral Hospital and Adama Health Center who visited these health institutions. Additionally, 21 MTBC species were isolated from suspected tuberculosis lesions in cattle slaughtered at the Adama municipal abattoir and Bishoftu ELFORA export abattoir. The isolates were identified at the strain and lineage levels based on spoligotyping. Analyzing strain diversity and comparing it with international databases is key to understanding the global distribution of *M. tuberculosis* strains. In this context, we described the strain diversity of *M. tuberculosis* complex among clinical isolates from pulmonary TB patients and from tuberculosis lesions in cattle slaughtered in central Ethiopia, and we compared these strains with those in the SITVIT2 database.

In the present study, culture positivity in primary culture media from animals was found in 26.3% (21/80) of tissue lesions suspected of TB. However, the isolation percentage of the *M. tuberculosis* complex from TB-suspected lesions was low in this study, similar to results from earlier studies (18, 21, 22). This low percentage of *M. tuberculosis* complex isolation in zebu cattle in Ethiopia could be associated with various factors. One potential factor is the containment of the *M. tuberculosis* complex within granulomas, which are usually calcified and could lead to the death of *M. tuberculosis*, resulting in a low yield from culturing these lesions. Over the last 2 decades, most lesions detected in zebu cattle have been localized to one anatomical site and calcified (14, 15, 22, 23). This low isolation rate of mycobacteria may also stem from reduced culture sensitivity due to prolonged storage at field sites, freeze–thaw cycles during transportation, contamination of tissue samples, and the overgrowth of *M. bovis* by environmental mycobacteria (24). Furthermore, the presence of caseous and/or calcified lesions, as well as lesions resembling tuberculous lesions, may not always indicate mycobacterial origin; they can be caused by other intracellular organisms or parasites, or viable mycobacteria may not be present in calcified lesions (13, 25).

In the present study, 21 animal isolates showed evidence of *M. tuberculosis* complex species, with 12 identified as *M. bovis* and nine as *M. tuberculosis*. Further characterization of the *M. bovis* strain through spoligotyping revealed the presence of 12 isolates grouped into nine clusters of spoligotype patterns. Of the nine clusters of *M. bovis*, seven were novel and had not been previously reported in the *M. bovis* spoligotype database, while the remaining two clusters corresponded to spoligotype SB1265 (two isolates) and SB1176 in the *M.bovis*.org database. Similar to this study, the spoligotype SB1265 has also been reported earlier (26). The isolation of SB1176 (*M. bovis*) indicates its dominance as a major cause of BTB in Ethiopian cattle (18, 23, 26–28). The spoligotype SB1176 carries a specific spoligotype feature (spacers 4–7 missing) typical of members of a clonal complex identified as *M. bovis* African 2 (Af2), which has so far only been found in East Africa (29).

The nine *M. tuberculosis* strains isolated from TB-suspected lesions in cattle belong to eight Euro-American lineages (SIT 50, 118, 117, and 1,318) and one Indo-Oceanic lineage. *M. tuberculosis* infection has been reported in a wide range of domestic and wildlife animal species, most frequently in those living in close, prolonged contact with humans (30–33).

Similar to the present study, the isolation of *M. tuberculosis* from TB-suspected lesions was reported in cattle in Ethiopia by others (18, 22, 34, 35). Moreover, *M. tuberculosis* has been sporadically isolated from cattle in other countries, as reported by (36). Animal attendants with active TB in the respiratory tract, urinary tract, or gastrointestinal tract also represent an active source of *M. tuberculosis* for animals, spreading the bacillus via sputum, urine, or feces (33). The isolation of *M. tuberculosis* from bovine tissues likely supports the possible transmission of *M. tuberculosis* from humans to cattle.

The isolation of *M. tuberculosis* from bovine tissues raises concerns about potential human-to-cattle transmission, although *M. tuberculosis* is primarily considered a human pathogen. Unlike *M. bovis*, the main causative agent of bovine tuberculosis with a well-established zoonotic cycle, *M. tuberculosis* typically lacks a known reservoir in cattle. However, previous studies have documented cases of *M. tuberculosis* infection in cattle, suggesting that close contact with infected humans, particularly in environments with poor biosecurity measures, may facilitate transmission (37, 38). Human-to-animal transmission of *M. tuberculosis* has been reported in other animals, indicating that cross-species transmission is possible under certain conditions (39, 40).

Possible routes of transmission to cattle include direct aerosol exposure in confined environments, ingestion of contaminated feed or water, and contamination during handling and milking. While such occurrences are rare, they highlight the need for improved surveillance of bovine tuberculosis cases that do not fit the classical *M. bovis* infection profile. Given the zoonotic potential of tuberculosis, distinguishing between *M. tuberculosis* and *M. bovis* infections in cattle is critical for understanding transmission dynamics and implementing appropriate control measures. Further molecular epidemiological studies are needed to confirm the origins of *M. tuberculosis* in cattle and to assess the risk of reverse zoonosis in agricultural settings.

Out of 392 positive sputum samples cultured, 86 (21.9%) were culture-positive. The culture positivity rate of sputum samples collected from TB-suspected human cases, such as those who have coughed for more than two weeks and experienced night sweats, was low. Similarly, an earlier study in Ethiopia reported a low percentage of *M. tuberculosis isolation* from the sputum of TB patients (41). This study indicated that 83.2% (247/297) of patients suspected of having pulmonary TB had a negative smear result for acid-fast bacilli. The likelihood of isolating the *M. tuberculosis* complex from smear-negative TB cases was low. The culture positivity of sputum is directly related to the presence of bacilli, which can also be affected by the nature of the granuloma. If the granuloma is dense and solid, the chances of bacilli excretion in the sputum are minimal, leading to a low yield from sputum culture. Additionally, if the sputum is not collected correctly or if its storage and processing for culture are not conducted according to optimal procedures, the yield of sputum culture will be low.

Spoligotyping of the 86 human isolates revealed 35 distinct spoligotype patterns, representing 40.7% of genotype diversity. The diversity of the spoligotype strains observed in this study was consistent with the 41.9% reported by others (42, 43), but higher than the percentages reported in earlier studies conducted in Ethiopia (44–46). This low diversity of spoligotype strains of *M. tuberculosis* could suggest that a limited number of lineages are responsible for the disease in the study area despite significant migrations of various infected ethnic groups to Adama City and its surroundings from other regions of the country.

A total of 65 human isolates were grouped into 14 clusters, resulting in an overall clustering percentage of 75.6%. The clustering rate observed in the present study was comparable to a national survey (47) and a study performed in the Afar pastoral region (42). However, the clustering rate reported in this study is higher than that found in earlier research (43, 48, 49). The observed differences in clustering rates might be related to variations in sanitation, housing, and population density. Additionally, a high level of strain clustering could suggest recent and ongoing TB transmission in the study area (50–52). Consequently, poor sanitation, poor housing, and high population density (overcrowding) facilitate TB transmission, thereby increasing the clustering rate, while adequate sanitation, proper housing, and low population density reduce TB transmission, resulting in a decreased clustering rate.

In the present study, the most commonly identified strains were SIT 149, SIT 53, and SIT 118, in order of decreasing frequency. Similar to this study, SIT149 was predominantly isolated in previous research conducted in the Afar Pastoral Region of Ethiopia (42). In accordance with the present study, SIT 149 (T3-ETH) is frequently isolated in Ethiopia and among Ethiopian immigrants in Denmark (20). A previous study in Ethiopia showed that SIT149 was the most frequently clustered strain among MDR-TB isolates (53). The second most common *M. tuberculosis* strain found in this study was SIT53, which has also been reported earlier in the SITVIT database as the most common type in Ethiopia by other researchers (54).

The strains of the *M. tuberculosis* complex isolated in the present study belonged to the *M. tuberculosis* species lineages: Euro-American, Indo-Oceanic, East-African-Indian, or the species *M. bovis* or *M. africanum* species. The dominant lineage was the Euro-American lineage, constituting 87.2% of the isolates. The Euro-American lineage is the most prevalent in the world (55) and in Ethiopia, ranging from 32.5% in Gambella near the South Sudan border to 86.8% (48) in central Ethiopia (56). It can be hypothesized that the Euro-American lineage may have been introduced to Ethiopia by Europeans during the Italian invasion. Screening of the SITVIT2 database also identified 3.5% (3/86) of the isolates as members of *M. africanum*. This finding is in line with the findings reported by (49). *M. africanum* has been recognized as a significant cause of human TB in West African countries (57, 58).

Among the 86 human isolates, 2 (2.3%) isolates were identified as *M. bovis*. The two human *M. bovis* isolates had identical spoligo patterns, suggesting that they originated from the same sources. The spoligotype is identical to SB1443 in the www.mbovis.org database. Additionally, the *M. bovis* isolates from human pulmonary TB patients matched the spoligotype of animal isolates (SB1443), which were previously identified in cattle from Nigeria (59). This finding suggests cattle-to-human transmission of *M. bovis*, as cattle are the primary hosts of this pathogen.

Humans can become infected with *M. bovis* through the ingestion of contaminated food from cattle or by inhaling particles from infected cattle. This finding is also supported by the findings of other studies (7), which indicate the involvement of *M. bovis* in TB, presumably linked to contact with infected cattle and the consumption of raw milk and meat (2). Overall, the contribution of *M. bovis* to TB in central Ethiopia was low (2.3%), similar to other studies conducted in Ethiopia.

In the present study, *M. bovis* was isolated from human TB patients, while on the other hand, *M. tuberculosis* was isolated from TB lesions in cattle. Thus, this study indicates that TB is a significant example of zoonosis and reverse zoonosis. Similarly, earlier studies reported the isolation of *M. bovis* from human TB patients in Ethiopia, although the percentage of isolation was low (27, 42, 49, 60, 61). Additionally, *M. tuberculosis* was isolated from TB lesions in cattle in Ethiopia and other countries (18, 36, 61–64). Moreover, *M. tuberculosis* was also isolated from various species of domestic and zoo animals (65–67). Although such studies are generally scarce in Africa, the isolation of *M. tuberculosis* has been reported from animals in a few African countries (68, 69). The possible sources of infection for animals with *M. tuberculosis* may include the sputum, urine, and feces of humans, arising from active cases of human TB in the respiratory tract, urinary tract, or gastrointestinal tract, respectively (33).

The present studies and earlier Ethiopian studies reported a higher percentage of *M. tuberculosis* (reverse zoonosis) isolation in cattle tissues than the percentage of *M. bovis isolation* in humans. This observation could suggest that reverse zoonosis is more important than zoonosis in terms of TB transmission in Ethiopia. However, people infected with *M. tuberculosis* may be more likely to seek medical attention, leading to an underestimation of the prevalence of *M. bovis* infection in the population.

The prevalence of bTB in indigenous cattle, which constitute approximately 98% of the Ethiopian cattle population, is low, resulting in a minimal chance of bTB transmission from cattle to humans. Conversely, Ethiopia is one of the 10 countries worldwide with a high burden of human TB (12), which increases the likelihood of *M. tuberculosis transmission* from humans to cattle due to the socio-cultural connections Ethiopian farmers maintain with their cattle. However, contrary to Ethiopian reports, studies from other African countries have indicated a higher percentage of *M. bovis isolation* from humans than the percentage of *M. tuberculosis isolation* from animals (7, 70–72).

## 5 Conclusion

This study indicated that both *M. tuberculosis* and *M. bovis* strains are circulating among humans and cattle in central Ethiopia. This finding highlights the challenges of TB control in the study area. Therefore, a One Health approach incorporating multidisciplinary control and preventive measures should be considered by the departments of public health, veterinary services, and environmental health in the study area.

## Data Availability

The datasets presented in this study can be found in online repositories. The names of the repository/repositories and accession number(s) can be found in the article/Supplementary material.

## Supplementary material

Supplementary IMAGE 1 Spoligotype pattern of mycobacteria isolated from humans. The profile is based on presence or absence of 43 spacers. No 1 and 43 show the patterns of positive control for M. tuberculosis; No 2 shows patterns of a negative control; No 3 show a positive control for Mycobacterium bovis. Patterns other than No 1, 2, 23 and 43 show the patterns of mycobacteria isolated from humans. Black squares show the presence of spacers while white dots show the absence of spacers.

Supplementary ImAGE 2 Spoligotype pattern of mycobacteria isolated from humans. The profile is based on presence or absence of 43 spacers. No 1 and 43 show the patterns of positive control for M. tuberculosis; No 2 shows patterns of a negative control; No 3 show a positive control for Mycobacterium bovis. Patterns other than No 1, 2, 23 and 43 show the patterns of mycobacteria isolated from humans. Black squares show the presence of spacers while white dots show the absence of spacers.

Supplementary IMAGE 3 Spoligotype pattern of mycobacteria isolated from animal and humans. The profile is based on presence or absence of 43 spacers. No 1 and 43 show the patterns of positive control for M. tuberculosis; No 2 shows patterns of a negative control; No 3 show a positive control for Mycobacterium bovis. Patterns other than No 1, 2, 23 and 43 show the patterns of mycobacteria isolated from humans. Black squares show the presence of spacers while white dots show the absence of spacers.
