# Supplementary figures and images for: Zoonotic transmission of the Mycobacterium tuberculosis complex between cattle and humans in Central Ethiopia
Source: Front Vet Sci. 2025 Mar 10;12:1527279. doi: 10.3389/fvets.2025.1527279 (PMC11931144; doi:10.3389/fvets.2025.1527279)

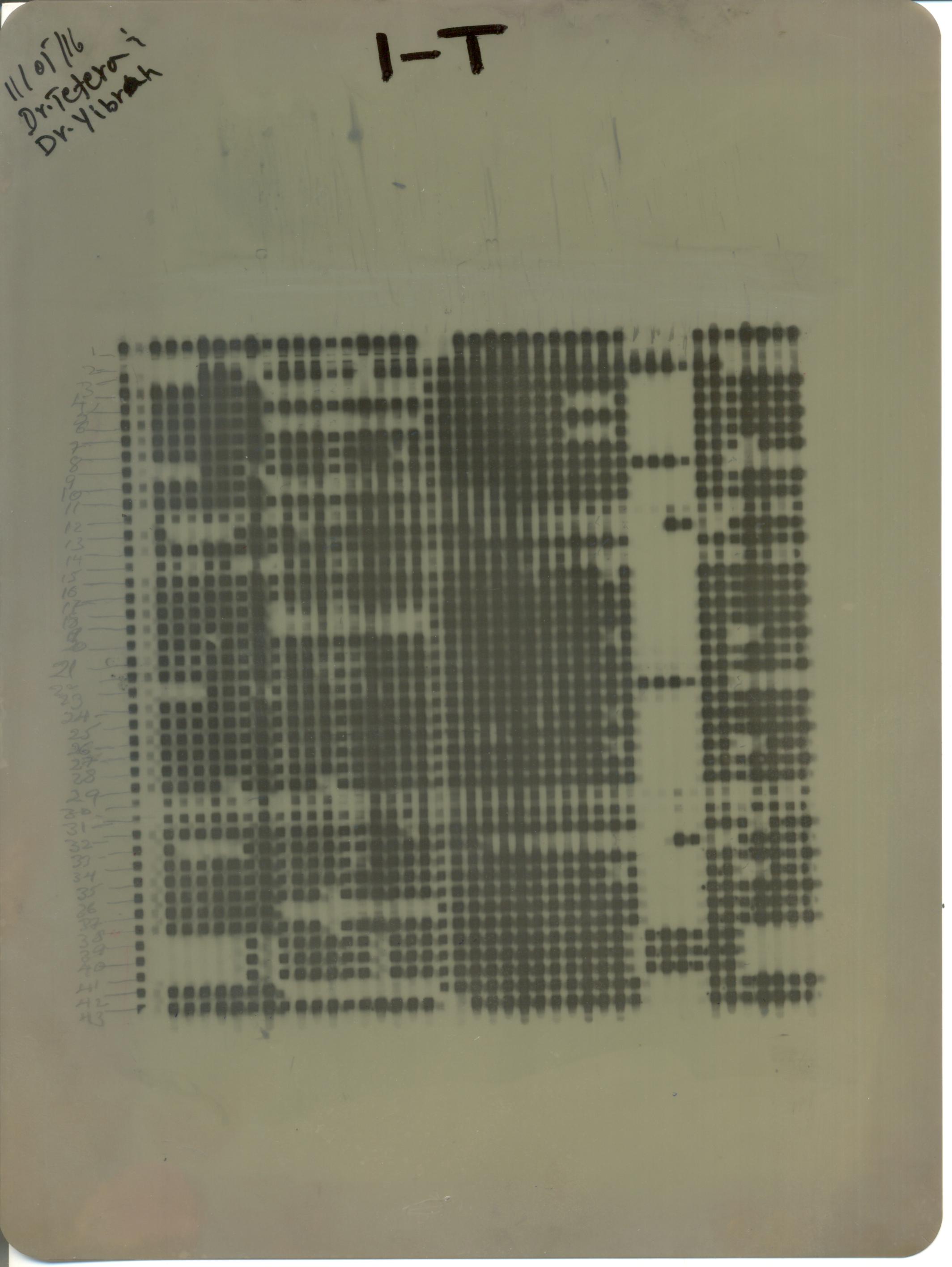

Supplement: Supplementary IMAGE 1 — Spoligotype pattern of mycobacteria isolated from humans. The profile is based on presence or absence of 43 spacers. No 1 and 43 show the patterns of positive control for M. tuberculosis; No 2 shows patterns of a negative control; No 3 show a positive control for Mycobacterium bovis. Patterns other than No 1, 2, 23 and 43 show the patterns of mycobacteria isolated from humans. Black squares show the presence of spacers while white dots show the absence of spacers. [file Image_1.jpeg]

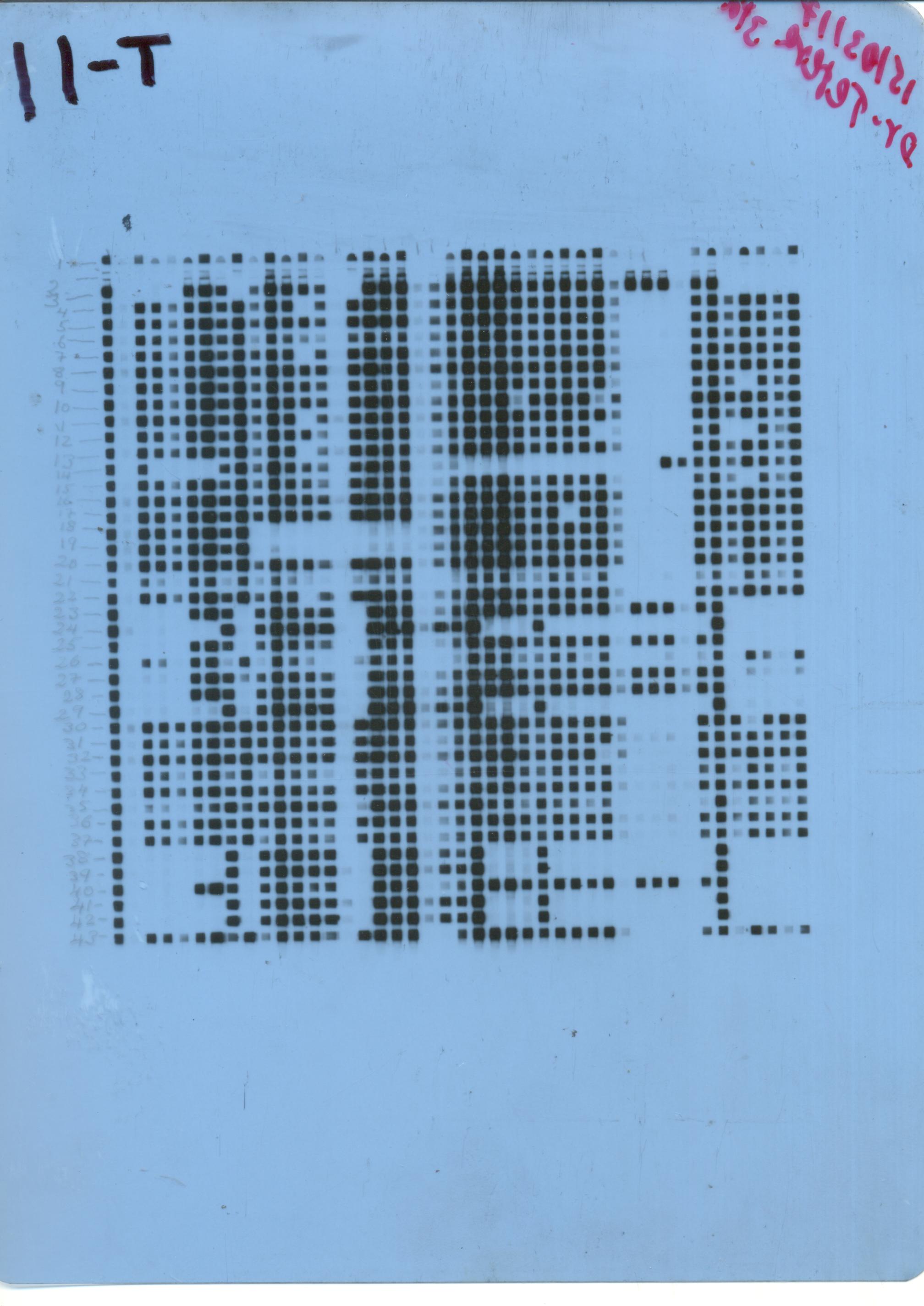

Supplement: Supplementary ImAGE 2 — Spoligotype pattern of mycobacteria isolated from humans. The profile is based on presence or absence of 43 spacers. No 1 and 43 show the patterns of positive control for M. tuberculosis; No 2 shows patterns of a negative control; No 3 show a positive control for Mycobacterium bovis. Patterns other than No 1, 2, 23 and 43 show the patterns of mycobacteria isolated from humans. Black squares show the presence of spacers while white dots show the absence of spacers. [file Image_2.jpeg]

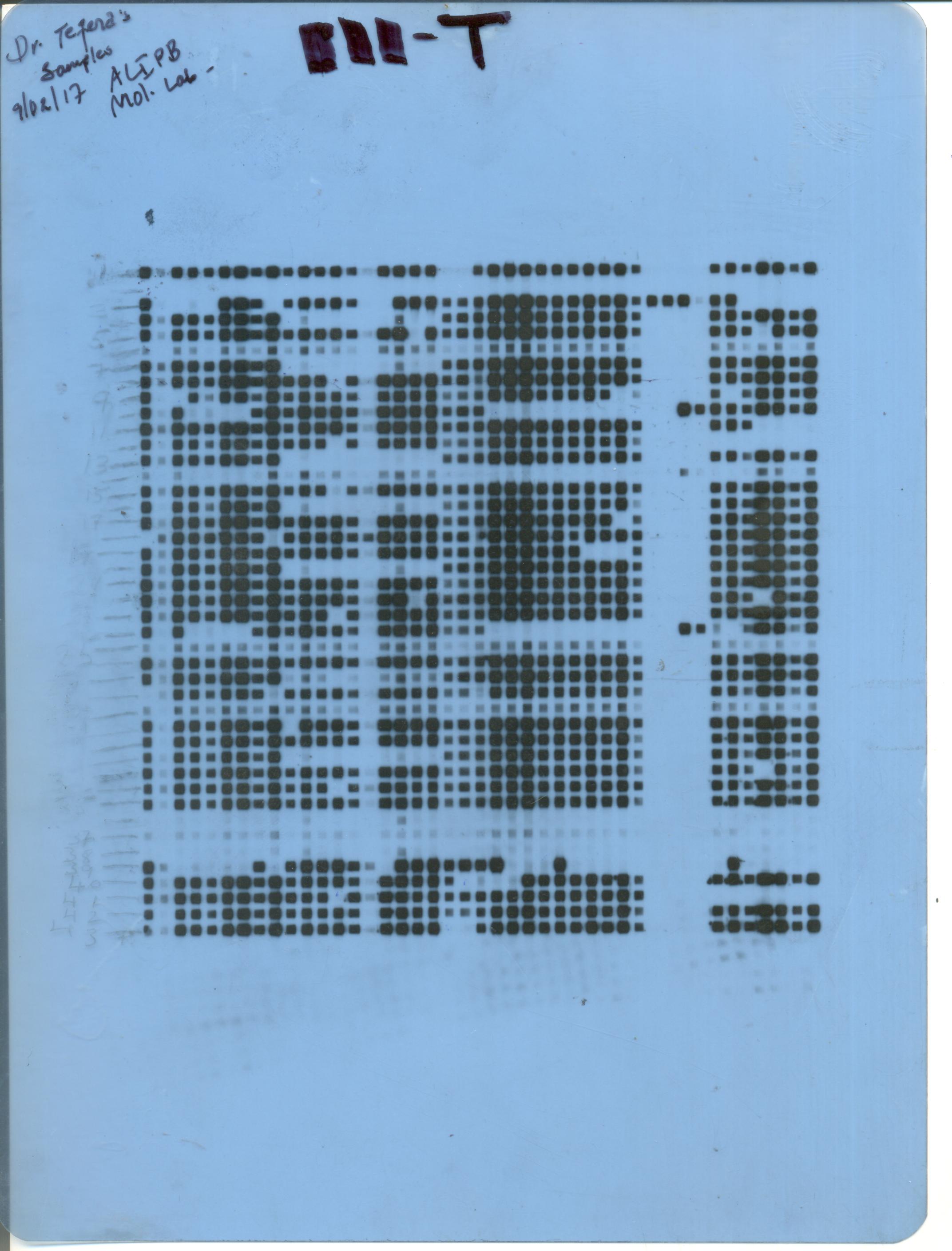

Supplement: Supplementary IMAGE 3 — Spoligotype pattern of mycobacteria isolated from animal and humans. The profile is based on presence or absence of 43 spacers. No 1 and 43 show the patterns of positive control for M. tuberculosis; No 2 shows patterns of a negative control; No 3 show a positive control for Mycobacterium bovis. Patterns other than No 1, 2, 23 and 43 show the patterns of mycobacteria isolated from humans. Black squares show the presence of spacers while white dots show the absence of spacers. [file Image_3.jpeg]
